# Timbe (*Acaciella angustissima*) as an Alternative Source of Compounds with Biological Activity: Antidiabetic

**DOI:** 10.3390/ph18040593

**Published:** 2025-04-18

**Authors:** Diana Karina Rangel-Sandoval, Lucia Guerrero-Becerra, Consuelo Lomas-Soria, Amanda Kim Rico-Chávez, José Antonio Cervantes-Chávez, Luis Antonio Reyes-Castro, Angélica Morales-Miranda, Ana Angélica Feregrino-Pérez

**Affiliations:** 1 Facultad de Ciencias Naturales, Universidad Autónoma de Querétaro, Campus Aeropuerto, Carretera a Chichimequillas s/n, Anillo Vial Fray Junípero Serra, Km 8, Querétaro 76000, Mexico; drangel11@alumnos.uaq.mx (D.K.R.-S.); jose.antonio.cervantes@uaq.mx (J.A.C.-C.); 2 Facultad de Ingeniería, Universidad Autónoma de Querétaro, Campus Amazcala, Carretera a Chichimequillas Km 1 s/n, Amazcala, El Marqués 76265, Mexico; gubel28@gmail.com; 3 Departamento de Biología de la Reproducción, Instituto Nacional de Ciencias Médicas y Nutrición Salvador Zubirán, Mexico City 14080, Mexico; consuelo.lomass@incmnsz.mx (C.L.-S.); luis.reyesc@incmnsz.mx (L.A.R.-C.); angelica.moralesm@incmnsz.mx (A.M.-M.); 4 Facultad de Química, Universidad Autónoma de Querétaro, Campus Centro Universitario, Cerro de las Campanas s/n, Querétaro 76010, Mexico

**Keywords:** antimicrobial activity, antioxidant activity, diabetes mellitus, hypertensive, legume, phenolic compounds

## Abstract

**Background/Objectives:** Timbe (*Acaciella angustissima*) is a legume recognized for its environmental benefits, such as soil restoration, wildlife nutrition, and the presence of biologically active compounds. This study investigates the antioxidant, pharmacological, and antimicrobial properties of Timbe. **Methods:** The total phenolic content, flavonoids, and condensed tannins from Timbe flowers, seeds, and pods were quantified, and their antioxidant activity was evaluated using the DPPH and ABTS assays. Enzymatic activities were assessed through α-amylase, α-glucosidase, and ACE-I inhibition, and antimicrobial properties were tested against various bacterial strains. **Results:** The pods and flowers exhibited higher antioxidant capacities compared to seeds, effectively neutralizing free radicals. Flavonoids and condensed tannins showed positive correlations with antioxidant activity and the inhibition of α-amylase and α-glucosidase, suggesting the potential benefits of these metabolites in blood glucose control. Timbe also demonstrated ACE-I inhibition, particularly the flowers. Regarding antimicrobial activity, the pods displayed moderate inhibition against *E. coli*, *K. pneumoniae*, and *S. aureus*. **Conclusions:** The results indicate that different parts of Timbe (flowers, seeds, and pods) possess significant therapeutic potential for preventing and treating metabolic disorders and bacterial infections.

## 1. Introduction

Traditional medicine encompasses knowledge and practices transmitted from generation to generation based on the experiences and beliefs of diverse cultures. It is essential in maintaining health and preventing diseases and is especially accessible in rural and remote areas [1]. Traditional medicine is based on the use of plants (seeds, roots, stems, leaves, flowers, and fruits) that contain bioactive compounds, such as secondary metabolites, which can also serve as precursors for designing new drugs, especially for chronic and degenerative diseases [2,3,4]. On the other hand, interest in medicinal herbs has grown in recent years because they usually cause fewer side effects than conventional drugs. The increase in the search for medicinal alternatives is valuable, particularly where modern and safe drugs do not exist, which is why the World Health Organization (WHO) recommends evaluating the effectiveness of plants in treating diseases [5,6,7]. Among bioactive plant metabolites, phenolic compounds are prominent because they are widely distributed in plants and have health-promoting effects [3,8]. The phenolic compounds found in many plant foods have a wide range of biological properties, including antioxidant, anti-inflammatory, and enzyme activity-modulating effects [9,10]. Although several processes through which phenolic compounds contribute to preventing metabolic diseases have been identified, the exact mechanisms by which they exert these effects are still under investigation [9]. However, growing evidence suggests that regular consumption may be an effective strategy for improving metabolic health and preventing the onset of diseases like diabetes mellitus (DM) [11,12].

DM is a chronic and complex metabolic disease characterized by elevated blood glucose levels and is common worldwide [13]. People with DM develop infections more frequently than the general population, and these tend to be more severe, increasing the risk of death, especially in low-income countries [14,15]. Infections, and in some cases, the treatments applied to them, can alter the balance of glucose in the body, affecting insulin production and effectiveness and increasing the risk of developing diabetes [14]. Additionally, modifying the diversity of the gut microbiota and gut-related metabolites is associated with beneficial effects that help combat diabetes [13,16]. The human gut microbiota, composed of trillions of bacteria from over 1000 species and other microorganisms such as archaea, viruses, fungi, and protists, plays a crucial role in health. Its imbalance or alteration can contribute to the development of various diseases, such as obesity, type 2 diabetes, nonalcoholic hepatic steatosis, hypertension, and osteoporosis. Due to the increasing prevalence of DM and its complications, natural plant-based treatments, such as legumes, are being investigated. Thanks to their bioactive compounds, they can regulate glucose levels and prevent metabolic complications [17].

Timbe (*Acaciella angustissima*) is a legume with a distribution ranging from the southern United States to Costa Rica, and it is adapted to dry and semi-arid ecosystems in Mexico. Its resistance to drought and ability to improve soil quality make it a key species for bioremediation and ecosystem restoration. Its morphological characteristics include taproot, compound leaves, white flowers, and pod-shaped fruits, and it is used to produce firewood, wood, fodder, and traditional medicinal products [18,19]. Timbe is valuable for its high protein content and ability to regrow, which makes it helpful as fodder in dry seasons. In addition, it has outstanding biological properties, such as antioxidant, antimicrobial, and antimutagenic activity, which gives it the potential for pharmacological and cosmetic applications [19,20,21]. The roots and bark are used in traditional medicine to relieve toothache, arthritis, gastritis, rheumatic disorders, and skin lesions and even to treat digestive problems and diarrhea [18,20]. Its effect on diseases such as diabetes and its ability to produce bioactive compounds give Timbe a promising biotechnological potential in the health sector. However, despite these benefits, Timbe has been threatened by deforestation, leading to the advent of reforestation and conservation initiatives [22,23]. Given its agroforestry importance, a deeper understanding of its value, including its bioactive potential, could strengthen sustainable utilization strategies. Therefore, this research aimed to identify and analyze the bioactive compounds present in the flowers, seeds, and pods of the Timbe (*Acaciella angustissima*) plant, not only to explore underexploited therapeutic applications but also to support conservation by highlighting its multifunctional role. This study focuses on the antioxidant, pharmacological (antidiabetic), and antimicrobial properties of the Timbe tree with a pioneering approach to exploring its bioactive compounds in the context of new therapeutic alternatives.

## 2. Results

### 2.1. Phenolic Compounds and Antioxidant Capacity

The analysis presented in Table 1 reveals significant differences in the content of bioactive compounds and antioxidant capacity between the flowers, seeds, and pods. The pods showed the highest concentration of total phenols (7.151 ± 0.04 mg GAE/g), with a statistically significant difference compared to the flowers and seeds, the latter having the lowest concentration. Regarding the flavonoid content, the flowers stood out with the highest value (4.052 ± 0.26 mg RE/g), while the seeds had the lowest concentration of these compounds. In the case of condensed tannins, the pods again stood out with the highest concentration (6.213 ± 0.64 mg CE/g), significantly surpassing the flowers and seeds, between which no significant differences were found. Regarding antioxidant capacity, the pods showed the highest value in the DPPH assay, followed by the flowers and, finally, the seeds, with the lowest activity. On the other hand, in the ABTS assay, the flowers and the pods showed significantly higher antioxidant activity than that observed for the seeds.

The antioxidant capacity of the samples (Table 1) was expressed in terms of the Trolox concentration that generates a similar decrease in absorbance compared to the reference sample. In the DPPH (2,2-diphenyl-1-picrylhydrazyl) assay, the pods showed the highest efficiency in neutralizing free radicals, with a value of 9.745 ± 0.07 mg Trolox/g, while the seeds presented the lowest antioxidant capacity, with a value of 3.979 ± 0.09 mg Trolox/g. In the ABTS (2,2′-azino-bis-3-ethylbenzothiazoline-6-sulfonic acid) assay, the flowers and pods showed higher antioxidant activity (8.261 ± 0.08 and 7.931 ± 0.08 mg Trolox/g, respectively) compared to the seeds, whose activity was significantly lower, reaching 5.989 ± 0.37 mg Trolox/g.

**Figure 1:**
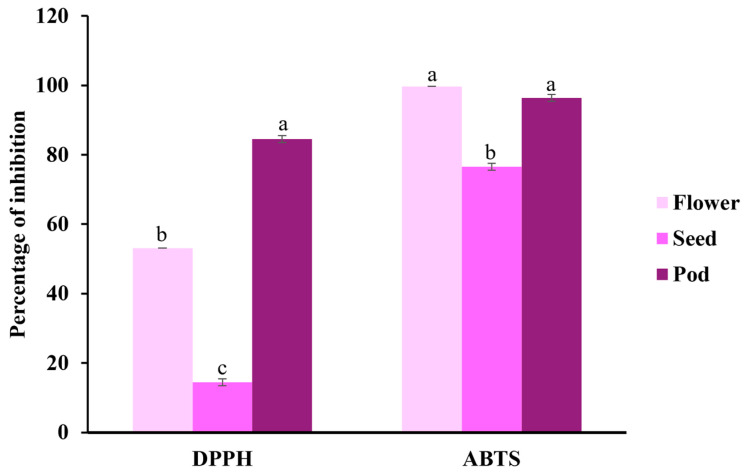
Percentage of inhibition of the antioxidant capacity of the flowers, seeds, and pods of *Acaciella angustissima.* Data are expressed as Mean ± SD; *p* < 0.05. A one-way ANOVA using Tukey’s test was performed to determine differences among tissues (flowers, seeds, pods) for each analysis. Different letters indicate statistical differences (*p* < 0.05).

Comparing the antioxidant activity from the ABTS and DPPH assays of the three samples, it can be observed that for the flowers, the ABTS method reaches almost 100% inhibition, while DPPH shows a value of 53.09%. In the seeds, both methods presented lower inhibition percentages than the flowers and pods, with DPPH being the lowest, with a value of 14.43%. Both methods show high inhibition percentages in the pods, reaching 96.36% and 84.52% (ABTS and DPPH, respectively), with ABTS slightly higher (Figure 1).

### 2.2. Metabolic Composition and Fatty Acid Content

The most relevant components of the metabolic profile of the flowers, seeds, and pods of *A. angustissima* were identified (Table 2) as the main metabolites in these parts of the plant, and their biotechnological potential was highlighted. D-pinitol and stigmasterol occur in the pods, seeds, and flowers (see footnote Table 2), while β-amyrin was predominantly in the flowers and pods; these compounds stand out for their potential biological properties.

The fatty acid profile (Table 3) revealed that most of the acids present in the samples are unsaturated fatty acids, predominantly in the seeds. However, it only contains two unsaturated fatty acids (oleic acid and linoleic acid), and their concentration is higher compared to the pods, which contain three unsaturated acids (6-Octadecenoic acid, linoleic acid, and 1,3,14,16-Nonadecatetraene). On the other hand, the flowers have the lowest concentration, with only linolenic acid present. The flowers stood out for their higher concentration of hexadecanoic acid. At the same time, the Timbe seeds contain a higher total amount of fatty acids, the most prominent being linoleic acid and oleic acid. Both were unsaturated acids, with the first being an essential fatty acid (omega 6) and the second an omega 9. The pods presented a great diversity of short-chain fatty acids, but the most abundant was 6-octadecenoic acid, although linoleic and palmitic acids were also present.

### 2.3. α-glucosidase, α-amylase, and ACE-I Inhibitory Activities

α-amylase, α-glucosidase, and ACE-I inhibition assays were carried out to determine the potential of Timbe flowers, pods, and seeds (concentration: 10 mg/mL) against diabetes and hypertension-related enzymes. The Timbe pods had a remarkable potential against enzyme activity related to diabetes and hypertension. In particular, they showed the most significant inhibitory activity for α-amylase, with 74.77%, compared to the flowers and seeds. However, regarding α-glucosidase inhibition, the pods showed the lowest inhibition, with 15.92%. On the other hand, the seeds presented the highest percentage of α-glucosidase inhibition, with 48.12%, followed closely by the flowers, with 47.72%. Notably, regarding ACE-I inhibition, lisinopril (reference standard) showed inhibition of 91.46 ± 4%, underscoring the importance of continuing to investigate the therapeutic potential of Timbe extracts for treating diabetes and hypertension to achieve the same percentages of inhibition as lisinopril, for example. The structure with the highest inhibitory activity was the flowers, whose extract showed 69.14% more inhibition than the pods and seeds (Table 4).

**Figure 2:**
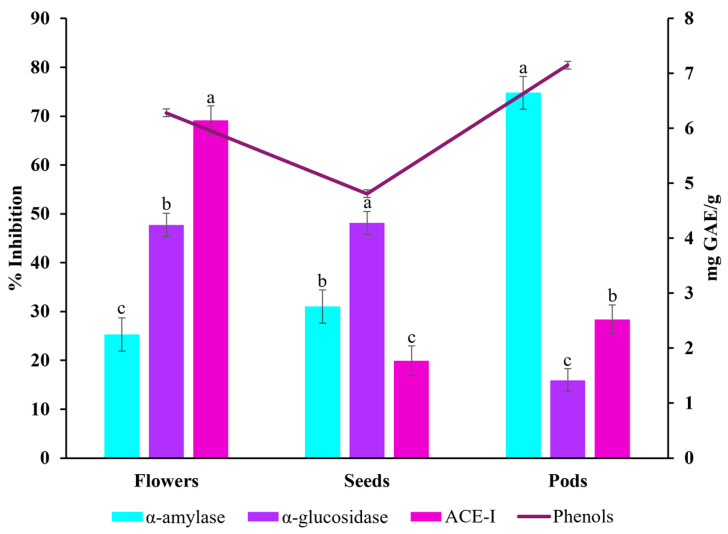
Comparison of the total phenolic content with the inhibitory activity of α-amylase, α-glucosidase, and ACE-I. Data are expressed as Mean ± SD. A one-way ANOVA followed by Tukey’s test was performed to determine differences among tissues (flowers, seeds, and pods) for each analysis. Different letters indicate statistical differences (*p* < 0.05). ACE-I: angiotensin I-converting enzyme inhibition; mg GAE/g: mg eq. gallic acid/g of sample.

A correlation analysis was carried out between the percentages of inhibition of the α-amylase, α-glucosidase, and ACE-I enzymes and the total phenolic content (Figure 2). The pods demonstrated both a high content of phenolic compounds and inhibitory activity, especially with α-amylase. Even though the seeds have the lowest phenolic content, they showed the highest percentage of inhibition for α-glucosidase. Nevertheless, the inhibition percentages of the flowers and seeds were not significantly different, which led to both having the highest inhibitory activity for α-glucosidase. The ACE-I inhibitory activity is the lowest in all the parts tested. In addition, the phenol levels follow a similar trend to enzyme inhibition, with higher values in the flowers and pods and lower values in the seeds.

**Figure 3:**
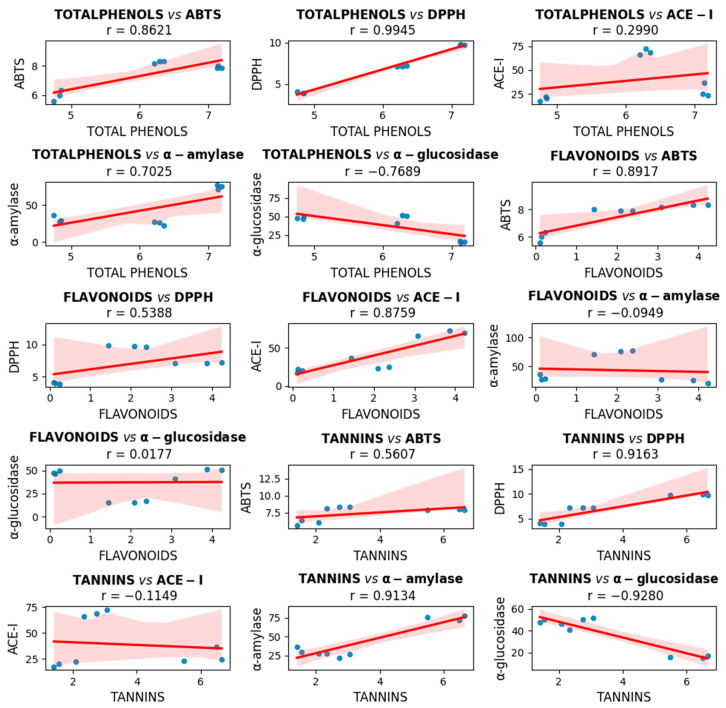
Correlation between total phenols, flavonoids, and tannins with DPPH, ABTS, α-amylase, α-glucosidase, and ACE-I (*p*-value < 0.05). Total phenols: mg eq. gallic acid/g of sample; flavonoids: mg eq. rutin/g of sample; condensed tannins: mg eq. (+) catechin/g of sample; DPPH, ABTS, α-amylase, α-glucosidase, and ACE-I: percentage of inhibition.

The correlation matrix between three types of bioactive compounds (total phenols, flavonoids, and tannins) and five biological activities (ABTS, DPPH, ACE-I, α-amylase, and α-glucosidase) is shown in Figure 3. Each graph presented a linear regression with its corresponding Pearson correlation value (r), which allowed the evaluation of the relationship between each pair of variables. Overall, the total phenols and tannins had strong positive correlations with the antioxidant activity (ABTS and DPPH), especially between phenols and DPPH (r = 0.99), suggesting a high antioxidant potential for these compounds. Furthermore, tannins showed significant negative correlations with α-glucosidase inhibition (r = −0.93) and positive correlations with α-amylase (r = 0.91), indicating a possible effect on enzyme modulation. On the other hand, flavonoids correlated well with ABTS and ACE-I but showed no clear relationship with digestive enzymes. Together, these results showed that certain phenolic compounds may have played a relevant role in antioxidant activity and the inhibition of enzymes associated with metabolic diseases, with additional details provided in Supplementary Material (Table S1).

### 2.4. Antimicrobial Activity

In this study, the MIC (minimum inhibitory concentration) values of the Timbe flowers, pods, and seeds against *Listeria monocytogenes*, *Staphylococcus aureus, Escherichia coli, Pseudomonas aeruginosa, Salmonella typhimurium*, and *Klebsiella pneumoniae* were determined by the microdilution method (Table 5). The extract dilutions considered were 20, 10, 5, 2.5, 1.25, 0.625, 0.312, 0.156, 0.078, and 0.039 mg/mL. The Timbe structures with the most prominent inhibitory activity were the pods against *S. aureus* and *K. pneumoniae*, showing the lowest MIC value at 0.625 mg/mL. These were followed by the seeds (MIC 1.25 mg/mL) and pods (MIC 1.25 mg/mL) against *S. aureus* and *E. coli*, respectively. Overall, the seeds showed the lowest performance regarding inhibitory effects against most of the bacteria tested, as the MIC obtained for most cases was ≥20 mg/mL, the highest concentration tested. The flowers presented a diverse spectrum of inhibition, ranging from MIC 2.5 mg/mL (against *S. aureus*) to MIC > 20 mg/mL (against *E. coli*).

## 3. Discussion

### 3.1. Antioxidant Capacity by Phenolic Compounds

*A. angustissima* is characterized by its remarkable resistance to extreme heat and drought conditions, which makes it an ecological and sustainable option in arid regions. In addition, these conditions favor the development of secondary metabolites (phenolic compounds, nitrogen compounds, sulfur compounds, and terpenes) that the plant produces as a defense mechanism. These compounds are relevant in both the chemical and pharmaceutical fields [19]. In the results presented, significant differences were observed in the concentrations of phenolic compounds between the different parts of the plant (flowers, seeds, and pods). These compounds were extracted using methanol from methanolic extracts using the technique employed in this study, which has proven to be more efficient compared to water extraction, as reported by Rodríguez-Méndez in 2018 [22]. The pods presented the highest concentration of phenolic compounds, with an average value of 7.151 ± 0.04 mg GAE/g, suggesting that this part of the plant could play a fundamental role in the storage or accumulation of these compounds as a strategy to protect the seeds [42,43]. The data obtained were lower than those that Rodriguez-Méndez et al. reported in a Timbe pod methanolic extract [22]. These variations could be attributed to the specific conditions in which the plant is found at the time of harvest since the threats faced by the plant can induce a greater production of secondary metabolites, particularly phenolic ones, which play a protective role [42,43,44].

On the other hand, the flowers showed the highest concentration of flavonoids, which contribute to the protection of the flowers from damage by pathogens and even from ultraviolet light. In addition, flavonoids give color to the flowers and attract pollinators, thus ensuring the perpetuation of the species and its continuity in the ecosystem [45]. Furthermore, the Timbe seeds presented the lowest amount of phenolic compounds, which agrees with that reported by Alonso-Herrera et al., who compared the seeds and the flowers [19]. These results could be explained by the distribution of the plant’s defense compounds, which are mainly concentrated in the parts most exposed to adverse environmental factors, such as the flowers and the pods, which protect the seeds from possible damage before germination [46].

Differences in the concentration of phenolic compounds between plant organs may be related to defense mechanisms optimized for their survival. Given its role in antioxidant capacity, this variation could help specifically counteract oxidative stress. Oxidative stress, caused by an imbalance between free radicals and antioxidants, is linked to chronic diseases. Genetic or environmental variations can influence antioxidant production, allowing some people to neutralize cellular damage more efficiently. Thus, these individual differences could offer more effective protection against the effects of oxidative stress. Dietary antioxidants, especially plant-derived polyphenols, help restore this balance by neutralizing free radicals by transferring a hydrogen atom, thus reducing cell damage [47,48]. A higher antioxidant capacity in a sample indicates a better ability to neutralize these free radicals, protecting cell health and preventing potential damage [49].

Among the parts of *A. angustissima* analyzed by ABTS, the flowers presented the highest concentration of Trolox equivalents, reaching almost 100% inhibition, suggesting a high efficacy against free radicals. In contrast, the seeds showed the lowest antioxidant capacity, reflected in the lowest percentage of inhibition. The results suggest that the antioxidant compounds present in the flowers react more efficiently with the ABTS radical than with DPPH. Both methods show the lowest inhibition percentages in the seeds, with DPPH showing the lowest value. This could be due to a lower concentration or effectiveness of antioxidant compounds in this part of the plant. In contrast, the highest inhibition values are observed in the pods for both methods, with ABTS showing a slightly higher percentage than DPPH. This behavior could be due to the presence of antioxidant compounds that are highly reactive against both radicals, although with a greater affinity towards ABTS, since a positive correlation has been seen between antioxidant activity with ABTS and DPPH, being more sensitive [50,51,52]. The relationship between antioxidant capacity and diabetes is that diabetic patients have greater oxidative stress due to high blood glucose levels, which can damage cells and tissues, contributing to complications such as cardiovascular problems, kidney damage, and neuropathy. Increasing antioxidant capacity helps neutralize free radicals generated by excess glucose, protecting cells and reducing the risk of complications [53,54,55].

Tannins act as effective electron and hydrogen donors, enabling them to neutralize free radicals and exhibit antioxidant activity. Their antioxidant potential depends on factors such as chemical structure and the oxidizing agent involved, with higher molecular mass generally enhancing activity. However, studies suggest that their effectiveness against DPPH radicals is primarily determined by the accessibility of hydroxyl groups [56]. On the other hand, there is a strong positive correlation between the concentration of flavonoids and their antioxidant capacity measured by ABTS. That is, the higher the content of flavonoids in a plant extract, the more significant the reduction in the ABTS radical and, therefore, the greater the measured antioxidant capacity [57,58]. A negative correlation between certain compounds, such as phenolics with α-glucosidase, flavonoids with α-amylase, and tannins with ACE-I and α-glucosidase, suggests that as the concentration of these compounds increases, their inhibitory capacity decreases. This could be due to competition with inhibitors at the enzyme’s active site or the activation of compensatory mechanisms that counteract the inhibition. Hyperglycemia favors oxidative stress since the autooxidation of glucose generates free radicals. Antioxidant capacity is crucial since hyperglycemia reduces this capacity, affecting various tissues and contributing to microvascular complications associated with diabetes. Improving erythrocytes’ antioxidant capacity and structure could prevent and treat such complications [59,60]. Polyphenols from plant sources have been associated with a lower incidence of metabolic diseases such as obesity, diabetes, and hypertension, which are related to insulin resistance [61]. These compounds improve insulin sensitivity, which is essential for people with diabetes. Furthermore, by reducing oxidative stress, polyphenols can prevent diabetes complications, such as blood glucose spikes and cardiovascular diseases, so their inclusion in the diet has positive effects [62,63,64]. Polyphenols may influence the activity of enzymes and hormones in the body, such as stimulating the release of GLP-1 (glucagon-like peptide-1), a hormone important for regulating blood sugar. GLP-1 and PYY (peptide tyrosine-tyrosine) reduce the risk of diabetes and obesity. They also modify the intestinal microbiota and may inhibit the enzyme DPP-IV (dipeptidyl peptidase-IV), which favors glucose control [65]. On the other hand, reactive oxygen species (ROS) are essential for regulating vascular and cardiac function. However, when their levels are elevated, they can cause cellular damage and contribute to the development of vascular disease, especially in conditions such as hypertension [66,67], which is a common complication of diabetes [68]. The flowers, seeds, and pods of *A. angustissima* presented a relevant antioxidant capacity attributed to their phenolic compounds. Phenolic compounds are known for their antioxidant properties, which help neutralize free radicals and prevent cellular damage, which could explain their ability to protect against mutations. This antioxidant activity is particularly relevant since, as Vargas-Hernández et al. point out, an extract with high phenolic content could protect DNA and minimize cellular damage [21]. However, although they show antimutagenic potential, evaluating their cytotoxicity is crucial to ensure that, at effective concentrations, they do not cause cellular damage.

### 3.2. Metabolic Profile and Fatty Acid Profile

The metabolic profiles of the flowers, seeds, and pods of *A. angustissima* were obtained by gas chromatography–mass spectrometry (GC-MS), revealing compounds with diverse biological activities. Three major compounds were identified in the flowers: D-pinitol (a sugar alcohol), stigmasterol (a sterol), and β-amyrin (a triterpene). Although only D-pinitol exhibits hydroxyl groups reminiscent of phenolics, all three are associated with antioxidant, anti-inflammatory, antidiabetic, anticancer, and immunomodulatory properties [27,28,29,30,31,32,33,34]. Moreover, phytosterols and triterpenes are known for modulating cellular processes and combating oxidative stress. Essential and non-essential amino acids, as well as myoinositol, were identified in the seeds, which are compounds with protective and regulatory effects on metabolic functions [35,36,37,38]. From the pods, picolinic acid and 2-ketoglutaric acid are reported to exhibit anti-inflammatory, antimicrobial, and prebiotic properties [41]. The presence of essential amino acids, phytonutrients, and antioxidant compounds, such as β-amyrin and picolinic acid, indicates that this plant may have protective properties, making it interesting from a pharmacological and nutritional perspective. The promising results suggest that the flowers, seeds, and pods contain a combination of bioactive metabolites that could benefit both the plant and humans.

Furthermore, the fatty acid profile found for the different parts of *A. angustissima* revealed a composition rich in compounds of nutritional and therapeutic relevance. The presence of saturated fatty acids, such as palmitic and stearic, along with unsaturated fatty acids, such as oleic, linoleic, and linolenic, suggested that this plant could play a beneficial role in human health, particularly in regulating cholesterol and cardiovascular function and modulating inflammatory processes. Polyunsaturated fatty acids, such as linoleic and linolenic, are essential, as the human body cannot synthesize them and must obtain them from the diet. They also play a key role in neuronal development, immune response, and the prevention of chronic diseases. The high concentration of these compounds in the pods, especially 6-octadecenoic acid, highlighted its potential as an alternative source of functional lipids. A comparison of the fatty acid profile of red lentil seeds shows similar results to those of Timbe seeds. Its main fatty acid is linoleic acid (46.81%), followed by oleic acid (23.27%) and palmitic acid (14.41%) [69,70], which shows a comparable trend to the content of the Timbe seeds, which presents values of 48.91%, 34.83%, and 12.12%, respectively. On the other hand, soy, another seed widely consumed for its nutritional value and health benefits [71,72,73], shows the same trend with higher concentrations of linoleic acid (52.8%), oleic acid (19%), and palmitic acid (13.1%) [74]. A moderate amount of linoleic acid in the diet may help lower total blood cholesterol levels and low-density lipoprotein cholesterol (known as “bad” cholesterol) [75].

The unique phytochemical profile of *A. angustissima*, featuring phenolic metabolites, sterols, and triterpenoids, positions it as a prime candidate for drug development and functional foods. Further research on the pharmacokinetics and structure–activity relationships of these compounds is warranted to unlock their full therapeutic potential.

### 3.3. α-glucosidase, α-amylase and ACE-I Inhibitory Activities

Type 2 diabetes is characterized by a rapid rise in blood glucose levels due to the breakdown of starch by pancreatic α-amylase and glucose absorption in the small intestine by α-glucosidase. This condition may be managed by inhibiting the enzymes involved in carbohydrate digestion. The consumption of natural inhibitors from dietary components could effectively control postprandial hyperglycemia with minimal side effects, unlike traditional drug treatments such as acarbose [76].

The effects of Timbe on enzymes associated with diabetes and hypertension have not been thoroughly investigated. However, it has been reported that in rats with streptozotocin-induced diabetes, Timbe (pods) significantly reduced blood glucose levels, increased serum insulin concentrations, decreased lipid levels, and improved indicators of kidney damage [22]. The present work assessed the inhibitory activity of α-glucosidase and α-amylase. The *A. angustissima* pod extract demonstrated the highest content of phenolic compounds and inhibitory activity of the α-amylase enzyme. In contrast, the seeds and flowers showed the most prominent inhibitory effect of the α-glucosidase enzyme. Therefore, all the studied structures of *A. angustissima* have potential as an alternative diabetes treatment. However, further studies are still required since none of the extracts evaluated achieved the percentage of inhibition reported in the standard, such as acarbose, which is 93% [77].

In addition, hypertension is one of the major macrovascular complications of diabetes and is a significant risk factor for many cardiovascular diseases. Hypertension is twice as common in people with diabetes as in those without. People with hypertension often have insulin resistance and are more likely to develop diabetes than people with normal blood pressure [78]. ACE-I plays a crucial role in the regulation of vascular tone. ACE-I converts angiotensin I into angiotensin II, a potent vasoconstrictor stimulating aldosterone secretion from the adrenal glands. ACE-I inhibition is an effective therapeutic approach for the treatment of hypertension in both diabetic and non-diabetic patients [79].

In this study, the inhibitory activity of ACE-I was evaluated. The flowers showed the best performance for ACE-I inhibition, making them the best candidate for treating hypertension of all the *A. angustissima* structures studied. Although the seeds and pods did not show as pronounced an inhibitory effect as the flowers, their activity on α-glucosidase and α-amylase was greater, making all structures relevant to the possible treatment and prevention of diabetes and hypertension. Plant extracts have been used worldwide as complementary or alternative treatments for patients with diabetes and hypertension. Their efficacy is largely attributed to the presence of polyphenolic compounds in various plants and foods, whose antioxidant activity helps neutralize free radicals and, consequently, prevent diabetes [80]. In addition, the hypoglycemic effects of these plants are also linked to the presence of compounds such as alkaloids, terpenes, flavonoids, and saponins, among others, which are thought to have insulin-mimetic activity. However, their exact mechanism of action remains unclear [22].

The correlation analysis showed that total phenols, flavonoids, and tannins exhibited varying degrees of association with the biological activities evaluated. These findings reinforce the importance of phenolic compounds as bioactive agents with multiple functional effects, especially in oxidative stress and metabolic diseases such as diabetes and hypertension.

### 3.4. Antimicrobial Testing

People with diabetes are more susceptible to new infections and relapses due to weakened immune defenses and complications of the disease. Rare, life-threatening infections are more common in people with diabetes than in those without diabetes [15].

Hyperglycemia, a key symptom of poorly controlled diabetes, plays a crucial role in exacerbating bacterial infections by providing an optimal environment for pathogen growth [14]. People with inadequate glycemic control have higher glucose levels in various tissues and organs throughout the body, which many bacterial pathogens, including staphylococci, streptococci, and enterococci, use as their primary carbon source to support their growth and enhance their virulence [81].

Plant-derived antimicrobials have immense potential for treating bacterial, fungal, protozoal, and viral infections without known adverse effects due to secondary metabolites, which include phenolic compounds such as flavones, flavanols, flavonoids, quinones, and tannins [82]. These are commonly found in medicinal plants and are widely used against pathogenic bacteria. *A. angustissima* is rich in phenolic compounds, which are effective in preventing a wide variety of diseases as they possess antidiabetic, antioxidant, anticancer, antimicrobial, and anti-inflammatory biological activities [18,83]. Timbe is also rich in flavonoids, which are particularly recognized for their antiviral, anti-inflammatory, and antimicrobial effects. Moreover, flavonoids exhibit promising activity against *E. coli*, *P. aeruginosa*, *K. pneumoniae,* and *Mycobacterium tuberculosis* [84,85]. Tannins have also been shown to inhibit the growth of several Gram-positive and Gram-negative bacteria and are known to have the ability to disrupt biofilms [86].

The antimicrobial activity of *A. angustissima* has been studied. Ethanolic extracts of this plant have shown a complete inhibition of growth on *Bacillus subtilis*, *Klebsiella pneumoniae*, and *Staphylococcus aureus* [87]. Methanolic extracts of pods have displayed antifungal activity against *Fusarium oxysporum, Rhizoctonia solani*, and *Phytophtora capsica,* as well as against the human pathogen *Candida albicans* [21]. In addition, methanolic extracts of pods have also demonstrated antifungal activity against *Sclerotium cepivorum* Berk in garlic cultivation [88]. Currently, there are many pharmacological treatment options for diabetic patients, but the associated side effects, such as gastrointestinal symptoms, heart failure, weight gain, edema, impaired renal function, pancreatitis, genital infections, etc., become an additional burden for patients [89]. Treatments with fewer side effects are needed, and plant extracts could be an effective therapeutic intervention.

The literature regarding the antimicrobial activity of *Acaciella angustissima*, or Acaciella as a genus, is limited, which makes interpreting results challenging. Nevertheless, several studies have been conducted concerning the Fabaceae family and reported the resulting minimal inhibitory concentration (MIC) and the different thresholds considered to classify the results. The MIC ranges considered to classify the antimicrobial activity of Fabaceae can vary vastly; in a study by Bussmann et al. [90] strong antimicrobial activity was defined as an MIC of less than 5 mg/mL, while other authors like Nielsen et al. [91], Dalmarco et al. [92], Tamokou et al. [93], and Wamba et al. [94] considered significant, excellent, or highly active when MIC < 0.1 mg/mL.

The difference in the thresholds used to classify the antimicrobial activity could be explained when looking at the MIC values obtained from plants from the Fabaceae family, as these also vary greatly. In the study by Bussmann et al. [90] it was shown that against *S. aureus*, *Caesalpinia paipai* and *Cassia fistula* showed an MIC value of 1 mg/mL; *Senna monilifera* and *Spartium junceum* of 4 mg/mL; *Caesalpinia spinosa* of 16 mg/mL; and *Senna bicapsularis* of 256 mg/mL. On the other hand, the MIC values for *E. coli* were 0.016 mg/mL for *Senna bicapsularis*, and 64 mg/mL for *Caesalpinia spinosa* and *Medicago sativa*. The MIC attained stand out for their high values, which might explain why strong antimicrobial activity was considered when MIC < 5 mg/mL.

Nielsen et al. [91] evaluated the antibacterial activities of the leaves and stems of *A. karroo,* obtaining an MIC value of 0.15625 mg/mL against βL+EC (β-lactamase-positive *Escherichia coli*) and CRPA (Carbenicillin-resistant *Pseudomonas aeruginosa*) for both leaf and stem extracts. In addition, the MIC values against MRSA (Methicillin-resistant *Staphylococcus aureus*) and ARKP (Ampicillin-resistant *Klebsiella pneumoniae*) were 0.15625 mg/mL and 0.07812 mg/mL in the case of leaves and stem, respectively. The activity was considered to be significant if the MIC values were below 0.100 mg/mL and moderate when 0.100  <  MIC  <  0.625 mg/mL.

The antimicrobial activity and MIC values of seeds and leaves of *Albizia masikororum* (Fabaceae) were analyzed in a study by Razafindrakoto et al. [95]. The MIC value against *S. aureus* was determined to be 0.19531 mg/mL and 0.78125 mg/mL for seeds and leaves, respectively. The MIC interpretation standards followed those established by Dalmarco et al. [92], where an MIC below 0.1 mg/mL was regarded as excellent, between 0.1 mg/mL and 0.5 mg/mL as moderate, between 0.5 mg/mL and 1 mg/mL as weak, and above 1 mg/mL as inactive.

A study by Mahamat Djamalladine et al. [96] analyzed the antimicrobial activity of the aerial parts of *Abrus canescens* (Fabaceae), which demonstrated notable antibacterial effects against *P. aeruginosa*, *E. coli*, *E. faecalis*, and *S. aureus*, with MIC values between 0.256 and 0.512 mg/mL. Another study by Álvarez-Martínez et al. [97] reviewed the antimicrobial activity of compounds derived from plants and their mechanisms of action in which some Fabaceae plants were assessed. The flowers of *Bauhinia kockiana* presented an MIC of 0.0625 mg/mL against Methicillin-resistant *S. aureus* (MRSA), and the leaves of *Phaseolus vulgaris* against *E. coli* had an MIC of 0.256 mg/mL. In both articles, the threshold values proposed by Tamokou et al. [93] were considered: highly active (MIC below 0.100 mg/mL), significantly active (0.100 ≤ MIC ≤ 0.512 mg/mL), moderately active (0.512 < MIC ≤ 2.048 mg/mL), low activity (MIC > 2.048 mg/mL), and not active (MIC > 10 mg/mL).

An experiment conducted by Mpude et al. [98] showed that the leaves of *Acacia sieberiana* were most active against Methicillin-susceptible *Staphylococcus aureus* (MSSA) A1 with an MIC of 0.032 mg/mL and against Methicillin-resistant *Staphylococcus aureus* (MRSA) A4 with an MIC of 0.128 mg/mL. The thresholds considered were significant activity (MIC < 0.1 mg/mL), moderate (0.1 mg/mL < MIC ≤ 0.625 mg/mL), and low or negligible (MIC > 0.625 mg/mL). Nevertheless, in this study is remarked the updated and rationally defined cutoff points of antibacterial botanicals obtained from Wamba et al. [94]: outstanding activity—minimal inhibitory concentration (MIC) ≤ 0.008 mg/mL; excellent activity—0.008 < MIC ≤ 0.04 mg/mL; very good activity—0.04 < MIC ≤ 0.128 mg/mL; good activity—0.128 < MIC ≤ 0.32 mg/mL; average activity—0.32 < MIC ≤ 0.625 mg/mL; weak activity—0.625 < MIC ≤ 1.024 mg/mL; not active—MIC values > 1.024 mg/mL.

Overall, the similarity between the threshold values for MIC proposed by Tamokou et al. [93] (considered by Mahamat Djamalladine et al. [96] and Álvarez-Martínez et al. [97]) and the ones proposed by Wamba et al. [94] (contemplated by Mpude et al. [98]) and by Dalmarco et al. [92] (considered by Razafindrakoto et al. [95]) creates a more solid and well-founded classification of the results for plants from the Fabaceae family.

Considering those ranges, the most prominent results obtained for *A. angustissima* come from the pods, with a weak or moderate antimicrobial activity against *S. aureus* (MIC 0.625 mg/mL) and *K. pneumoniae* (MIC 0.625 mg/mL). Both bacterial strains are relevant to common diabetes infections: *S. aureus* is recurrent in infections like pneumonia and bronchopneumonia, infections in skin lesions, necrotizing fasciitis, diabetic foot, osteomyelitis, septic arthritis, and bacteremia [15,99]; *K. pneumoniae* has been linked to endophthalmitis, pneumonia, bronchopneumonia, and hepatic and intra-abdominal abscesses in people with diabetes [15]. *K. pneumoniae* is also known to cause urinary tract infections in diabetic patients, which often become more severe due to immunosuppression [100,101].

The inhibition of *E. coli* due to the pods (MIC 1.25 mg/mL), as well as the inhibition caused by the seeds against *S. aureus* (MIC 1.25 mg/mL), can also be classified as moderately active if just the classification by Tamokou et al. [93] is considered. Although the pods did not show the best inhibitory effect of this experiment, it stands out from the rest as its inhibition towards *E. coli* (MIC 1.25 mg/mL) surpassed that of gentamicin (MIC 20 mg/mL). *E. coli* is also a microorganism responsible for common infections in people with diabetes, such as endophthalmitis, bacteremia, sepsis, and urinary tract infections (UTIs) like cystitis, urethritis, and pyelonephritis [15].

The inhibition of the rest of the tested bacteria caused by the seeds and pods was not enough to classify them in an excellent, strong, high, or even a moderate category, receiving a lower classification just like for the flowers, which are classified as inactive, low activity, or negligible as none of the concentrations against any of the tested bacteria reached an MIC value low enough (lowest MIC = 2.5 mg/mL against *S. aureus*). Even though the antimicrobial activity against the rest of the tested bacteria was not as prominent as the ones already described, their potential should not be ignored as other common infections in people with diabetes are caused by *Salmonella* spp. (necrotizing fasciitis [15]), *P. aeruginosa* (malignant otitis externa, bacteremia, sepsis [15], and tissue damage in diabetic foot ulcers [102]), and *L. monocytogenes* (the combination of incomplete Freund’s adjuvant (IFA) and *L. monocytogenes* treatment effectively delays type I diabetes in non-obese diabetes (NOD) mice [103]).

## 4. Materials and Methods

### 4.1. Collection of Plant Material

In 2022, the flowers and mature pods were collected from *Acaciella angustissima* trees at the Autonomous University of Querétaro, Amazcala Campus, in El Marqués, Querétaro. The flowers were collected in July, and after selection, they were frozen for subsequent freeze-drying. The pods collected in February were left to dry at room temperature for 72 h; then, the seeds were separated from the pods for later handling. The samples were ground and sifted.

### 4.2. Content of Total Phenols, Flavonoids, and Condensed Tannins

To determine phenolic content, methanolic extracts were prepared following the methodology of Cardador-Martínez et al. [104] with some modifications. A total of 200 mg of the sample was weighed, and 10 mL of methanol was added. The mixture was subjected to sonication for 30 min at room temperature, and then centrifuged at 8000 rpm for 20 min at 4 °C. The supernatant obtained was recovered and stored at −20 °C until further use.

The total content of phenolic compounds was determined by the Folin–Ciocalteu method reported by Oomah et al. [105], one of the most used techniques for quantifying phenolic compounds due to its high sensitivity and reproducibility. For the standard curve, gallic acid was used (points between 0 and 0.032 mg/mL). The methodology proposed by Oomah et al. [68] was also followed to determine the flavonoid content, with a routine standard curve with points ranging from 2 to 200 mg/mL. Regarding the analysis of condensed tannins, the vanillin-HCl method was used according to the procedure described by Deshpande and Cheryan [106], with a Catechin standard curve within a 0 to 1 mg/mL concentration.

### 4.3. Antioxidant Capacity: DPPH Y ABTS

#### 4.3.1. DPPH

The DPPH (2,2-diphenyl-1-picrylhydrazyl) radical scavenging capacity was evaluated using the methodology described by Brand-Williams et al. [107], with some modifications. A total of 20 µL of the sample (methanolic extract described in Section 4.2) and 200 µL of a methanolic solution (80%) of DPPH (0.06 mg/mL) were added. The mixture was allowed to stand at room temperature for 30 min in the dark. Methanol was used instead of the DPPH solution for the blank, and deionized water was used instead of the sample for the control. The absorbance was measured at 515 nm. The free radical scavenging capacity was calculated using Equation (1).

%Free radical scavenging = [1 − (*A*_sample_ − (*A_blank_*/*A_control_*))] ∗ 100 (1)

Several points with concentrations ranging from 0 to 0.250 mg/mL of Trolox were used for the standard curve.

#### 4.3.2. ABTS

The ABTS (2,2′-azinobis-(3-ethylbenzothiazoline-6-sulfonic acid)) radical scavenging capacity was determined following the methodology described by Re et al. [108] with some modifications. A solution of ABTS radical (3.84 g/mL) was prepared with potassium persulfate (2.45 mmol/L), and it was left in the dark at room temperature for 12 h. A total of 20 µL of the sample (methanolic extract described in Section 4.2) and 230 µL of ABTS solution were added. For the blank, 20 µL of methanol plus 230 µL of ethanol were used, and for the control, only methanol was used. The absorbance was measured at 734 nm. The free radical scavenging capacity was calculated using Equation (2).

%Free radical scavenging = [1 − (A_sample_/*A_control_*)] ∗ 100 (2)

The standard curve was constructed using different points ranging from 0 to 0.250 mg/ml Trolox concentrations.

### 4.4. Analysis by Gas Chromatography–Mass Spectrometry (GC-MS)

#### 4.4.1. Fatty Acid Profile

To determine fatty acids, 50 mg of the sample (ground and sieved) was weighed and 400 μL of NaOH (1.5 M, prepared in methanol) was added, following the methodology of Gómez-Velázquez et al. [109]. The mixture was vortexed for 1 min and then sonicated at 40 kHz for 5 min. Then, 400 μL of H_2_SO_4_ (1.75 M, prepared in methanol) was added, vortexed again for 1 min, and sonicated for 5 more minutes. A total of 800 μL of hexane was added to each sample, vortexed for 30 s, and centrifuged at 10,000× *g* for 5 min to recover the supernatant. This was filtered and injected into the gas chromatograph.

#### 4.4.2. Metabolic Profile

The metabolic profile of the *A. angustissima* flowers, seeds, and pods was determined using 50 mg samples (ground and sieved), following the methodology of Rico-Chávez et al. [110]. Each sample was added with 1 mL of methanol, sonicated for 15 min, and then vortexed for 30 s. They were kept under constant agitation at 200 rpm in an orbital shaker for 3 h. The samples were centrifuged at 12,000 RCF for 10 min at 4 °C. A 250 μL aliquot was taken from the supernatant and dried in a SpeedVac at room temperature. For the derivatization of the samples, 100 μL of derivatizing agent (BSTFA + 1% TMS) was added, sonicated for 3 min, and centrifuged at 10,000× *g* for 5 min to recover the supernatant, which was then filtered to be injected into the gas chromatograph.

### 4.5. Evaluation of Enzymatic Activity

#### 4.5.1. Extract Preparation

The flower, pod, and seed extracts of *A. angustissima* were prepared using different solvents. For the α-amylase and α-glucosidase assays, distilled water was used at concentrations of 50 mg/mL and 20 mg/mL, respectively [79]. Methanol was used as the solvent at 1 mg/mL per extract for the ACE-I assay [111]. The samples were mixed and stirred at 160 rpm for 24 h in the dark at room temperature [112]. The homogenates were then centrifuged at 10,000 rpm at 4 °C for 20 min, and the supernatant obtained was used as the extract [113].

#### 4.5.2. α-amylase Inhibition Assay

A modified version of the assay described in the Worthington Enzyme Manual was used to evaluate the α-amylase inhibitory activity [79,114,115]. A 0.02 M sodium phosphate buffer (pH 6.9 with 0.006 M NaCl) was prepared to suspend the α-amylase solution (0.5 mg/mL) and a 1% starch solution. In total, 500 µL of each extract and the α-amylase solution were mixed and incubated at 25 °C for 10 min. Then, 500 µL of the 1% starch solution was added and incubated at 25 °C for another 10 min. The reaction was stopped by adding 1.0 mL of DNS dye reagent, and the tubes were boiled for 5 min, cooled, and diluted with 15 mL of distilled water. The absorbance was measured at 540 nm, and the blank and control values were recorded. The percentage inhibition was calculated using Equation (3) [113].

%Inhibition = [(A_control_ − A_sample_)/(A_control_)] ∗ 100 (3)

#### 4.5.3. α-glucosidase Inhibition Assay

For α-glucosidase inhibitory activity, a modified version of the assay described in the Worthington Enzyme Manual [79,115,116] was used. A total of 50 µL of the sample extract, diluted with 50 µL of 0.1 M potassium phosphate buffer (pH 6.9), and 100 µL of 0.1 M potassium phosphate buffer (pH 6.9) were incubated with α-glucosidase solution (1.0 U/mL) at 25 °C for 10 min. Then, 50 µL of a 5 mM solution of *p*-nitrophenyl-α-D-glucopyranoside in 0.1 M potassium phosphate buffer (pH 6.9) was added and incubated at 25 °C for 5 min. A control with 50 µL buffer solution was included instead of the extract (A405 control). Absorbance readings were taken at 405 nm, and the percentage inhibition was calculated using Equation (3) [113].

#### 4.5.4. ACE-I Assay

The inhibition of angiotensin I-converting enzyme (ACE-I) activity was evaluated using the method established by Hou and collaborators [117], with modification [111]. All the tests were performed in triplicate. A Tris-HCl solution (50 mM, NaCl 0.3 M, pH = 7.5) was prepared as a solvent for an N-[3(2-furyl)acryloyl]-phe-gly-gly solution. First, 200 µL of this solution was mixed with 250 µL of the extract and 30 µL of Tris-HCl buffer. The enzymatic reaction was started by adding 20 µL of the ACE-I solution derived from rabbit lungs (1 U/mL) and later monitored for 5 min at 345 nm using a UV-Vis spectrophotometer. A blank containing no substrate was used as a control, while Lisinopril (2 mg/mL) was the positive inhibition control. For inhibition calculations, the enzyme activity without extract was considered to be 100%.

### 4.6. Assessment Antimicrobial Activity

#### 4.6.1. Extract Preparation

Methanol was used as a solvent to extract bioactive compounds from Timbe flowers, pods, and seeds. A total of 5 g of each Timbe structure was weighed and mixed separately with 500 mL of methanol. The mixtures were shaken in an orbital shaker at 160 rpm for 24 h at room temperature in the dark. After extraction, the methanol was evaporated using a rotary evaporator [118]. The resulting powder was dissolved in Mueller–Hinton Broth to obtain a 20 mg/mL concentration.

#### 4.6.2. Microorganisms and Growing Conditions

Six types of microorganisms were obtained from the collection of the Molecular Microbiology Laboratory from the Basic and Applied Microbiology Unit, Natural Sciences Faculty of the Autonomous University of Queretaro for the evaluation of the antibacterial activities of the Timbe flower, pod, and seed extracts. *Listeria monocytogenes* ATCC 19115, *Staphylococcus aureus* ATCC 25923, *Escherichia coli* ATCC 25922, *Pseudomonas aeruginosa* ATCC27853, *Salmonella typhimurium* ATCC 14028, and *Klebsiella pneumoniae* ATCC 13883 were standardized to a concentration of 10^8 CFU/mL for the antibacterial susceptibility test.

#### 4.6.3. Broth Microdilution Method

To evaluate the antimicrobial activity, a microdilution assay was performed to determine the minimum inhibitory concentrations (MICs) following the Clinical and Laboratory Standards Institute (CLSI) guidelines [119].

Serial dilutions of gentamicin (80 mg/2 mL) and the extracts at different concentrations (20, 10, 5, 2.5, 1.25, 0.625, 0.312, 0.156, 0.078, and 0.039 mg/mL) prepared in Mueller–Hinton Broth (MHB) and 50 µL of each dilution was transferred into a 96-well microtiter plate. The inoculants were standardized to 10^8 CFU/mL and diluted to 10^6 CFU/mL. A total of 50 µL of the diluted bacterial suspension was added to each well, resulting in a final concentration of 5 × 10^5 CFU/mL [120]. Positive controls (inoculants) and sterility controls (culture media) were considered [121]. The microplates were then incubated at 37 °C, and the wells were examined for turbidity 24 h later by optical density readings at 600 nm and by the direct observation of turbidity. At least three repetitions were run for each assay [122]. The lowest concentration of each extract that inhibited each bacteria’s growth was considered the MIC.

### 4.7. Statistical Analysis

All the tests were performed in triplicate, and the results were analyzed using a one-way ANOVA analysis followed by Tukey’s test. Significant differences between treatments were considered when the *p*-value < 0.05. All the statistical analyses were performed using the JMP Pro-16.2.0 software. To explore possible correlations between variables (total phenols, flavonoids, condensed tannins, ABTS, DPPH, ACE-I, α-amylase, and α-glucosidase), Python 3.13.2 was used together with the Pingouin library 0.5.5. This tool allowed for calculating pairwise correlations between variables using the Pearson method.

## 5. Conclusions and Perspectives

*Acaciella angustissima* is a promising alternative in biotechnology and health due to its bioactive properties. The flowers, pods, and seeds exhibit distinct phenolic compound content, correlating to their antioxidant, antidiabetic, and antihypertensive potential. The pods stood out for their high phenolic compound content, tannin content, and antioxidant capacity in the DPPH assay. At the same time, the flowers, the main source of flavonoids, showed the highest antioxidant activity in ABTS. In contrast, the seeds showed the lowest values in all the analyses, indicating a lower antioxidant potential. These results underline the therapeutic value of the plant, particularly in the regulation of blood glucose levels through the inhibition of alpha-amylase (with the pods being the most effective) and alpha-glucosidase (with the flowers and seeds being the most effective), in addition to its antihypertensive action through ACE-I inhibition (with the flowers having the greatest inhibition). These effects make *Acaciella angustissima* an attractive option for developing natural therapeutic products. Regarding antimicrobial activity, the pods showed the greatest inhibitory effect on bacteria, such as *Escherichia coli*, *Klebsiella pneumoniae*, and *Staphylococcus aureus*, suggesting their potential as natural agents for controlling bacterial infections. These results open the door for future research to explore their application in developing new antimicrobial treatments.

However, despite the promising results, further research is needed to explore the full therapeutic potential of the plant. Future studies should focus on understanding the specific mechanisms by which the bioactive compounds exert their effects, assessing the bioavailability and safety of the active constituents, and conducting clinical trials to confirm their efficacy in humans. In addition, research into the synergistic effects of their compounds could lead to more effective formulations for treating diseases such as diabetes and hypertension. A key area of future research will be to evaluate how these compounds affect the body’s homeostasis, particularly in the regulation of insulin secretion, which could offer new approaches to the treatment of metabolic disorders. Exploring sustainable extraction methods and expanding their applications in pharmaceuticals and nutraceuticals could also enhance their commercial potential and consolidate them as a valuable resource in natural medicine.

## Figures and Tables

**Table 1:** Content of phenolic compounds and antioxidant capacity of flowers, seeds, and pods of *Acacciella angustissima.*

Analysis	Flowers	Seeds	Pods
Total Phenols (mg GAE/g)	6.281 ± 0.07 ^b^	4.810 ± 0.05 ^c^	7.151 ± 0.04 ^a^
Flavonoids (mg RE/g)	4.052 ± 0.26 ^a^	0.121 ± 0.02 ^c^	2.235 ± 0.20 ^b^
Condensed Tannins (mg CE/g)	2.714 ± 0.36 ^b^	1.677 ± 0.36 ^b^	6.213 ± 0.64 ^a^
DPPH (mg Trolox/g)	7.160 ± 0.02 ^b^	3.979 ± 0.09 ^c^	9.745 ± 0.07 ^a^
ABTS (mg Trolox/g)	8.261 ± 0.08 ^a^	5.989 ± 0.37 ^b^	7.931 ± 0.08 ^a^

Data are expressed as Mean ± SD; *p* < 0.05. A one-way ANOVA followed by Tukey’s test was performed to determine differences among tissues (flowers, seeds, and pods) for each analysis. Different letters indicate significant statistical differences (*p* < 0.05) among the flowers, seeds, and pods for each phenolic compound and antioxidant capacity. mg GAE/g: mg eq. gallic acid/g of sample; mg RE/g: mg eq. rutin/g of sample; mg CE/g: mg eq. (+) catechin/g of sample; mg Trolox/g: mg eq. Trolox/g of dry sample.

**Table 2:** Metabolic profile of *Acaciella angustissima* flowers, seeds, and pods obtained by gas chromatography coupled with mass spectrometry (GC-MS).

Sample	Retention Time (min)	Name	Area (%)	Associated Biological Activity
Flowers	7.200	L-Proline	2.724	Precursor to the synthesis of compounds with antioxidant and antidiabetic potential [24,25].
	12.491	L-Threonic acid	1.607	In the form of magnesium salt, it could have beneficial neurofunctional effects in the treatment of Attention-deficit hyperactivity disorder [26].
	17.489	D-Pinitol	5.528	It has been found in other fabaceous, such as soybeans and tamarind, and is credited with antidiabetic, anti-inflammatory, antioxidant, and immunosuppressive properties [27,28,29].
	39.682	Stigmasterol	6.905	It has anticancer, anti-inflammatory, antioxidant, neuroprotective, antidiabetic, and antiparasitic effects, as well as anti-osteoarthritis, immunomodulatory, antifungal, and antibacterial properties [30,31,32].
	40.441	β-Amyrin	11.324	It has antioxidant potential, anti-inflammatory effects, antimicrobial activity, and antidiabetic properties [33,34].
Seeds		Amino acid		Amino acids can act as precursors in the synthesis of antibacterial and anticancer agents, while non-essential amino acids can modulate and enhance antitumor function [35,36,37].
	5.759	L-Valine	0.694	
	6.741	L-Leucine	0.126	
	7.202	L-Proline	0.187	
	8.456	Serine	0.107	
	8.987	L-Threonine	0.115	
	9.595	L-Aspartic acid	0.216	
	19.652	D-pinitol	0.502	It is the same compound as in flowers.
	21.697	Myo-Inositol	1.289	It shows protective effects against oxidative damage in proteins and lipids, improving vascular function and blood coagulation [38].
	39.678	Stigmasterol	0.247	It is the same compound as in flowers.
Pods	11.515	Picolinic acid	2.565	Picolinic acid derivatives have demonstrated antitumor, antiangiogenic, and antimicrobial effects [39,40].
		D-pinitol	8.083	It is the same compound as in flowers.
	19.367	2-Ketoglutaric acid	1.953	It acts as a prebiotic that reduces inflammation, improves the function of the intestinal barrier, and restores the balance of the intestinal microbiota [41].
	39.678	Stigmasterol	13.063	It is the same compound as in flowers.
	40.438	β-Amyrin	3.282	It is the same compound as in flowers.

**Note:** Only compounds whose presence or concentration are directly related to the activities analyzed in this study are reported. Other compounds present in the sample were not included in the table.

**Table 3:** Fatty acid profile of *Acaciella angustissima* flowers, seeds, and pods obtained by gas chromatography coupled with mass spectrometry (GC-MS).

Sample	Retention Time (min)	Name	Area (%)	Concentration (µg/g Sample)
Flowers	14.011	Hexadecanoic acid	58.54	334.329
	16.711	Stearic acid	13.69	125.154
	20.451	Linolenic acid	25.99	215.076
	22.739	Popenoic acid	1.78	NQ
Seeds	14.017	Palmitic acid	23.07	843.249
	16.676	Stearic acid	4.02	237.454
	17.408	Oleic acid	17.10	2424.105
	18.700	Linoleic acid	55.31	3404.042
	20.490	Eicosanoic acid	0.48	50.888
Pods	8.858	Ethanedioic acid	0.76	NQ
	9.439	Propanedioic acid	0.32	NQ
	10.194	Butanedioic acid	0.54	NQ
	12.130	Tetradecanoate acid	1.67	ND
	13.997	Palmitic acid	37.87	498.944
	14.436	Butanoic acid	0.64	NQ
	15.712	Nonanedioic acid	0.60	ND
	16.640	Stearic acid	12.05	260.789
	17.313	6-Octadecenoic acid	20.17	1033.730
	18.667	Linoleic acid	23.06	511.773
	20.532	1,3,14,16-Nonadecatetraene	2.29	42.122

NQ: not quantified; ND: not detected (concentrations were under the detection limit).

**Table 4:** Evaluation of enzymatic activity: α-amylase, α-glucosidase, and ACE-I.

	% Inhibition		
Assay	Flowers	Seeds	Pods
α-amylase	25.31 ± 3 ^b^	31.01 ± 4 ^b^	74.77 ± 3 ^a^
α-glucosidase	47.72 ± 6 ^a^	48.12 ± 2 ^a^	15.92 ± 1 ^b^
ACE-I	69.14 ± 3 ^a^	19.95 ± 2 ^b^	28.39 ± 7 ^b^

Data are expressed as Mean ± SD; *p* < 0.05. A one-way ANOVA followed by Tukey’s test was performed to determine differences among tissues (flowers, seeds, and pods) for each analysis. Different letters indicate significant statistical differences (*p* < 0.05) among the flowers, seeds, and pods. ACE-I: angiotensin I-converting enzyme inhibition.

**Table 5:** Minimum inhibitory concentration (MIC) of *Acaciella angustissima* flowers, seeds, and pods.

Bacteria	Minimum Inhibitory Concentration (mg/mL)			
	Gentamicin	Flowers	Seeds	Pods
*S. typhimurium*	0.039	5	>20	5
*E. coli*	20	>20	>20	1.25
*P. aeruginosa*	0.039	5	20	5
*S. aureus*	0.039	2.5	1.25	0.625
*K. pneumoniae*	0.039	10	>20	0.625
*L. monocytogenes*	0.039	5	>20	5

## Data Availability

The original contributions presented in this study are included in the article/Supplementary Material. Further inquiries can be directed to the corresponding author.

## Supplementary Materials

The following supporting information can be downloaded at: https://www.mdpi.com/article/10.3390/ph18040593/s1, Table S1. Person correlation for total phenols, flavonoids, tannins, DPPH, ABTS, α-amylase, α-glucosidase, and ACE-I (*p*-value < 0.05).
