# Supplementary material for: Timbe (Acaciella angustissima) as an Alternative Source of Compounds with Biological Activity: Antidiabetic
Source: Pharmaceuticals (Basel). 2025 Apr 18;18(4):593. doi: 10.3390/ph18040593 (PMC12030449; doi:10.3390/ph18040593)
Supplement: Supplementary file 1 [file pharmaceuticals-18-00593-s001.zip › pharmaceuticals-3593331-supplementary.pdf]

Supplementary Material

Table S1. Person correlation for total phenols, flavonoids, tannins, DPPH, ABTS, α-amylase, α-glucosidase, and ACE-I (p-value < 0.05).

X	Y	r	CI95%	p-unc	BF10	Power
Total phenols	ABTS	0.862	[0.46, 0.97]	$2.80x10^{-3}$	18.178	0.911
Total phenols	DPPH	0.994	[0.97, 1.0]	$4.11x10^{-8}$	$6.06x10^{-4}$	1.000
Total phenols	ACE-I	0.298	[-0.46, 0.8]	$4.35x10^{-1}$	0.533	0.124
Total phenols	α-amylase	0.702	[0.07, 0.93]	$3.48x10^{-2}$	2.824	0.608
Total phenols	α-glucosidase	-0.768	[-0.95, -0.21]	$1.55x10^{-2}$	5.116	0.739
Flavonoids	ABTS	0.891	[0.56, 0.98]	$1.24x10^{-3}$	33.312	0.952
Flavonoids	DPPH	0.538	[-0.2, 0.89]	$1.34x10^{-1}$	1.090	0.340
Flavonoids	ACE-I	0.875	[0.51, 0.97]	$1.96x10^{-3}$	23.656	0.931
Flavonoids	α-amylase	-0.094	[-0.71, 0.61]	$8.08x10^{-1}$	0.417	0.055
Flavonoids	α-glucosidase	0.017	[-0.65, 0.67]	$9.64x10^{-1}$	0.407	0.049
Tannins	ABTS	0.560	[-0.16, 0.89]	$1.16x10^{-1}$	1.203	0.370
Tannins	DPPH	$9.16x10^{-1}$	[0.64, 0.98]	$5.14x10^{-4}$	63.891	0.977
Tannins	ACE-I	$-1.15x10^{-1}$	[-0.72, 0.59]	$7.69x10^{-1}$	0.422	0.059
Tannins	α-amylase	0.913	[0.63, 0.98]	$5.78x10^{-4}$	58.551	0.975
Tannins	α-glucosidase	-0.928	[-0.99, -0.69]	$3.07x10^{-4}$	93.451	0.986

Number of observations: 9; r: correlation coefficient; 95%CI: 95% confidence interval for r;p-unc: p-value of the significance test; BF10: Bayes factor; Power: statistical power of the correlation test.
